# Surgical Therapy of Cervical Spine Fracture in Patients With Ankylosing Spondylitis

**DOI:** 10.1097/MD.0000000000001663

**Published:** 2015-11-06

**Authors:** Jun Ma, Ce Wang, Xuhui Zhou, Shengyuan Zhou, Lianshun Jia

**Affiliations:** From the Department of Spine Surgery, Changzheng Hospital, Second Military Medical University, Shanghai, China.

## Abstract

The present study aimed to explore surgical treatments and assess the effects based on the features of cervical spine fracture in patients with ankylosing spondylitis (AS) and to summarize the experiences in perioperative management. Retrospective analysis was performed in 25 AS patients with cervical spine fracture treated in our hospital from January 2011 to December 2013. The patients were divided according to fracture segments, including 4 cases at C4 to C5, 8 cases at C5 to C6, and 13 cases at C6 to C7. Among them, 12 belonged to I type, 5 to II type, and 8 to III type based on the improved classification method for AS cervical spine fracture. The Subaxial Cervical Spine Injury Classification score for these patients was 7.2 ± 1.3, and the assessment of their neurological function states showed 6 patients (24%) were in American Spinal Injury Association (ASIA) A grade, 1 (4%) in ASIA B grade, 3 (12%) in ASIA C grade, 12 (48%) in ASIA D grade, and 3 (12%) in ASIA E grade. Surgical methods contained simple anterior approach alone, posterior approach alone, and combined posterior–anterior or anterior–posterior approach. The average duration of patients’ hospital stay was 38.6 ± 37.6, and the first surgical methods were as follows: anterior approach alone on 6 cases, posterior surgery alone on 9 cases, and combined posterior–anterior or anterior–posterior approach on 10 patients. The median segments of fixation and fusion were 4.1 ± 1.4 sections. Thirteen patients developed complications. During 2 to 36 months of postoperative follow-up, 1 patient died of respiratory failure caused by pulmonary infections 2 months after leaving hospital. At the end of the follow-up, bone graft fusion was achieved in the rest of patients, and obvious looseness or migration of internal fixation was not observed. In addition, the preoperative neurological injury in 12 patients (54.5%) was also alleviated in different levels. AS cervical spine fracture, an unstable fracture, should be treated with operation, and satisfactory effects will be achieved after the individualized surgical treatment according to the improved classification method for AS cervical spine fracture.

## INTRODUCTION

Ankylosing spondylitis (AS) is a chronic inflammatory spondyloarthropathy featured by sacroiliitis and axial joint lesions. With the progression of AS, the ligaments, zygapophysial joints, and intervertebral discs are gradually ossified, which will result in spinal bony rigidity with the changes of bamboo spine. Meanwhile, fractures can occur under slight forces or without force because of the increased vertebral osteoporosis and bony brittle.^1^ Cervical spine is the position where spinal fractures frequently occur in AS patients.^2^ As for cervical spine fracture in AS patients, its therapeutic methods are extraordinary because it possesses different pathological characteristics of involved cervical spine, pathogenesis of fractures, clinical features, and prognosis compared with general cervical spine fractures.^3^

Nonsurgical methods for cervical spine fracture in AS patients include axial traction,^4^ halo vest, or cervicothoracic bracing treatments.^5–7^ Surgery treatment is the traditional therapy for cervical spine fracture in AS patients. The purpose of the treatment is to decompress the spinal cord, reduce dislocation, and exterminate vertebral mobilization. Anterior plating, posterior plates fixed with lateral mass screws,^8^ and posterior wiring technique are the main surgical treatments. Indeed, cervicothoracic bracing and axial traction treatments have not been widely used in clinic, due to high frequency of complications and death.^9^ Meanwhile, halo vest treatment was also associated with complications.^10^ Compared with nonsurgical treatments, surgical treatments appear to produce immediate stability and avoid the need of prolonged bed rest for cervical traction and immobilization. However, Lü et al^11^ found that anterior implant cannot resist the tension from the posterior spinal column. Meanwhile, Cooper et al^12^ demonstrated the failure of posterior fusion without the anterior support. The combined anterior and posterior surgery is regarded as an effective methods due to its low implant failure and high spinal recovery.

The present article retrospectively analyzed the characteristics of cervical spine fractures in 25 AS patients admitted during January 2011 and December 2013, and evaluated the surgical strategies. All relevant information were recorded below.

## MATERIALS AND METHODS

### Ethics Statement

This study was approved by the Changzheng Hospital ethics committee.

### General Data

A total of 25 patients (aged 34–89), 21 males and 4 females, with a median age of 56.9 ± 15.6 included in this study. In terms of injury mechanisms, 16 patients had hyperextension injury caused by falling on ground, 4 people by traffic accident, 3 by high falling, and 2 by crashing. Among these patients, 4 cases had fracture segments at C4 to C5, 8 cases at C5 to C6, and 13 people at C6 to C7.

**FIGURE 1:**
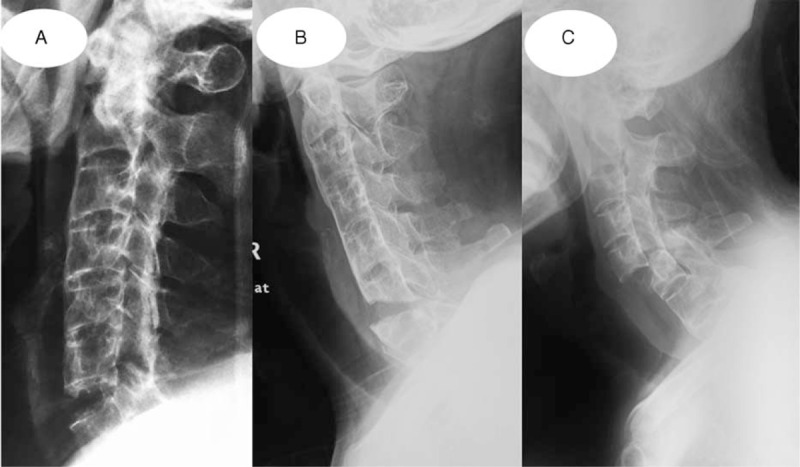
Improved AS cervical fracture classification: (A) I type with separated fracture passing intervertebral discs without obvious dislocation; (B) II type with separated fracture traversing vertebral body without obvious displacement; and (C) III type with blowout fracture across intervertebral discs or/and vertebral body with remarkable dislocation. AS = ankylosing spondylitis,.

According to the classification method offered by Caron et al,^13^ AS cervical spine fracture can be divided into the following types: A type refers to fractures threading intervertebral discs, B type means those through vertebral body, C type indicates those with anterior part across vertebral body while posterior part passing intervertebral discs, and D type presents those with anterior part passing intervertebral discs but posterior part traversing vertebral body. Based on the understanding of the features of AS cervical spine fracture, the operators improved the above classification method in certain degrees, and the modified classification included 3 types: I type refers to separated fractures passing intervertebral discs without obvious dislocation, II type stands for separated fractures through vertebral body without obvious displacement, and III type presents blowout fractures traversing intervertebral discs or/and vertebral body with obvious migration (Fig. 1). The patients in this study constituted of 12 cases with I type, 5 cases with II type, and 8 cases with III type in accordance with the improved classification method.

Subaxial Cervical Spine Injury Classification (SLIC) System proposed by Spine Trauma Study Group in 2007 has been initially verified in clinic to be effective in therapeutic guiding.^14–16^ This system graded on injury severity with a total score of 10 on the basis of 3 components, namely the injury morphology (0–4), the integrity of disco-ligamentous complex (0–2), and neurological function states (0–4). The treatment options are as follows: recommending conservative treatment if the overall score of SLIC is not >3,and surgical therapy when the score is not <5. The SLIC score for the patients before operation in our study was 5 to 9 with an average score of 7.2 ± 1.3, indicating all patients met the standards for surgical treatment.

As for cervical spinal cord injury scale proposed by American Spinal Injury Association (ASIA) before operation, 6 cases (24%) were classified into ASIA A grade, 1 (4%) into B grade, 3 (12%) into C grade, 12 (48%) into D grade, and 3 (12%) into E grade. The changes of patients’ neurological function states were constantly observed during treatment and follow-up.

## THERAPEUTIC METHODS

### Preoperative Preparation

All patients after hospitalized were assessed their following systemic conditions: nutritional status like serum albumin index; respiratory functions: blood gas analysis and respiratory function test; injured cervical spinal cord segments and ASIA grading of neurological function, as well as their ability of cough and expectoration; respiratory management: strengthening phlegm reduction through turning over and patting back at regular times. As for patients with obvious pulmonary infections, it was necessary to determine whether tracheotomy and assisted respiration with ventilator are needed, and sputum suction was performed with fiberobronchoscopy on the day of the surgery; combined injury such as traumatic brain injury, contusion, and abdominal visceral injury; and basic diseases, including cardiovascular disease, diabetes, and kidney disease. Preoperative preparation was carried out through focal adjustment on patients after the assessment for early surgery under permitting conditions. The patients were divided according to their fracture types through the modified classification method for AS cervical spine fracture. All III type patients adopted skull traction before operation, and pulley devices were placed above the head to ensure traction direction and neck in a straight line with a traction weight of 4 to 6 kg for 5 to 7 days due to anterior flexion of these patients.

### Operative Method Selection

The operators improved the classification method for AS cervical spine fracture proposed by Caron based on the understanding of the characteristics in AS cervical spine fracture, and determined surgical approaches for each patient in accordance with their fracture types. Anterior approach, as the first choice in I type fracture above C6/C7, cleaned fractured intervertebral discs and cartilage endplates, and then completed bone grafting fusion and anterior cervical plate fixation with at least 2 segments fixed both upper and lower. As for I type fracture at C6/C7, posterior bone grafting fusion and internal fixation was adopted when there were difficulties in anterior long segment fixation with plate. In II type, as the anterior approach applying cross-segment fixation makes it difficult to ensure the soundness in fixation, posterior bone grafting fusion and internal fixation was performed with at least 2 segments fixed both upper and lower. For those patients with excessive separation in anterior fractures or obvious anterior compression, anterior approach was applied for fracture gap decompression bone grafting, and for anterior plate fixation if necessary based on patients’ vertebral body bone. III type patients received conventional skull traction following hospitalization and x-ray examination of lateral cervical vertebrae with traction beside bed before operation. Patients were treated with corresponding surgeries according to the methods for I or II type if their fracture dislocations were reset. If not, there usually existed posterior locked facet, posterior approach as the first choice was operated for the restoration of spinous process after removing the locked facets under traction, for the fixation of each 2- to 3-segment lateral mass up and down or vertebral pedicle screw, and for bone grafting fusion between vertebral laminae. At last, anterior approach wasused for fracture gap decompression bone grafting, and anterior plate fixation was chosen when necessary depending on the state of patients’ vertebral body bone.

## RESULTS

### Clinical Outcome

The hospital stays of all patients were from 8 to 130 days with a median duration of 38.6 ± 37.6. All operations saw a success, and the operative time ranged from1.5 to 8.4 hours with an average of 4.2 ± 2.1 hours. The bleeding volume in surgery was 150 to 1900 mL with a mean quantity of 1008 ± 559 mL. In first surgery, 6 and 9 cases were merely undergone anterior approach and posterior approach, respectively, whereas combined surgery of posterior–anterior or anterior–posterior was applied on 10 cases with an average segments involving fixation and fusion of 4.1 ± 1.4 (whether the combined surgery belonged to posterior–anterior or anterior–posterior was determined by its more fixed segments using posterior or anterior approach). During postoperative follow-up of 2 to 36 months, 1 patient died of respiratory failure because ofo pulmonary reinfection 2 months after leaving hospital. By the end of the follow-up, the bone grafting fusion was achieved in the rest of patients; meanwhile, internal fixation showed no remarkable loosening or displacement. The severity of neurological function injury in 12 cases (54.5%) before surgery was alleviated to different degrees (Table 1). Three subjects of Frankle A were presented, less than that of preoperation (6). Patients of Frankle D was reduced after surgical treatment (12 vs 7). Meanwhile, 10 patients of Frankle E were observed after the treatment, more than that of preoperation (3).

**TABLE 1:**
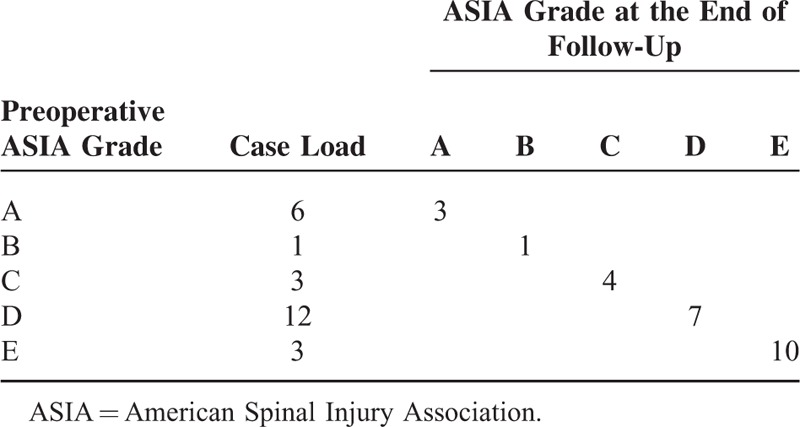
ASIA Grading for Neurological Function Before Operation and at the End of Follow-Up Respectively Among 25 Patients

### Complications

**FIGURE 2:**
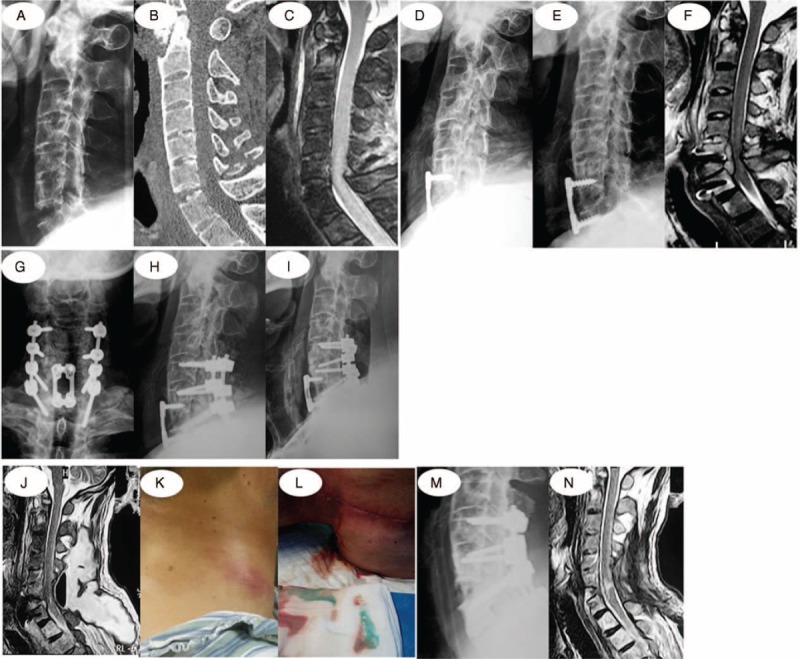
Main symptoms of a 49-year-old male patient suffering from cervical spine hyperextension injury caused by falling on ground with numbness and weakness. (A–C) X-ray and CT examination results show synostosis in each section of cervical spine with fracture separation at C6/C7, which belongs to I type, whereas MRI displays compression and edema in spinal cord. (D) In 2 segments fixed and fused with anterior approach, titanium plates and screws are well located at the end of the operation. (E, F) Five months after the operation, the patient expressed progressive neurological deterioration accompanied with internal fixation having slight dislocation shown by x-ray; besides, MRI examination reveals edema in prevertebral soft tissues, abnormal signals in C6/C7, high signals occupying posterior vertebral body, and spinal cord compressed. (G, H) Patient's neurological function was satisfactorily restored after posterior revision surgery. (I–L) Four months after revision operation when the patient developed fever coupled with mass in his right neck. X-ray and MRI examinations show swollen prevertebral soft tissues and gas in abscess cavity behind the neck, and these syndromes were confirmed as esophagus leakage by green dyestuff-like fester mixed in abscess cavity behind the neck after oral MeiLan. (M, N) Any leakage of contrast media could not be found after the oral omnipaque after 12 weeks of the following treatments: removing anterior cervical titanium plates and screws in second surgery, incision debridement, gastric intubation, enteral nutrition, and incision open drainage; in addition, abscess cavity, pneumatosis, or obvious abnormal signal shadow of soft tissues was not observed through MRI. All these indicate that esophagus leakage was healed. CT = computed tomography, MRI = magnetic resonance imaging.

Complications occurred in 13 cases. Two patients, having loosening anterior screws in reexamination, received posterior revision operation in our hospital 3 and 5 months later, respectively, after the first anterior surgery of 2 segments fixation and fusion operated in other hospitals. Four cases developed progressive neurological deterioration. Among 8 patients with pulmonary inflection, 4 underwent tracheotomy during hospitalization and successful incision blocking before leaving. Six patients had urinary track infection, and 2 with bedsores were basically cured by dressing change when out of the hospital. The following descriptions were the detailed information about 3 patients with esophagus leakage: one of them, confirmed on the fourth day after operation, was considered to have esophageal injury caused by fracture displacement or intraoperative traction; 2 were confirmed through oral Meilan imaging, gastrointestinal barium radiography, or electronic gastroscopy 9 and 16 months later, respectively, after operation when they readmitted because of the original symptom featuring fever coupled with shoulder masses. In the other 2 patients, 1 had esophagus abrasion caused by anterior cervical spine screw loosening 8 months after operation, whereas the other one (16 months later) was injured by repeating and long-term compression on posterior esophagus from thyroid cartilage during deglutition. After treatment, the patient with early-onset esophagus leakage was healed perfectly 4 weeks later, whereas for those with late-onset leakage, one (16 months after operation) of them was completely cured 6 weeks later and the other (9 months after operation) was 12 weeks later. Figure 2 lists a typical case.

## DISCUSSION

With the increase in spinal bone rigidity, vertebral osteoporosis, and bone brittle, AS patients often adopt head forward-bending position, causing progressive cervical kyphosis.^17–19^ AS cervical spine fracture is different from general traumatic cervical spine fractures because of its unique pathological characteristics as follows: AS cervical spine fracture tends to be caused by light traumas, even those not felt^20^; hyperextension injury represents the most common injury mechanism; this unstable fracture often affects anterior, middle, and posterior column with high dislocation probability; the major position lies in C5 to C7, and the fracture line ordinarily passes intervertebral discs; and the incidence rate is high in terms of spinal neurological function damage and complications. Sixteen patients (64%) in our study had ground-fall-caused cervical hyperextension injury mainly occurring at C5 to C7 (84%) with 13 people (52%) at C6 to C7. Meanwhile, 22 cases (88%) got neurological function injury at different levels. Especially, as AS cervical spine fracture prefers occurring on lower cervical spine, lateral x-ray may be not able to manifest fracture displacement due to the blocking of shoulder, tending to misdiagnosis. Therefore, AS patients with cervical spine injury should receive routine cervical spine computed tomography and magnetic resonance imaging examinations.

Conservative treatments for AS cervical fracture include cervical collar fixation, skull traction, and halo external fixation.^21,22^ These treatments, however, have been shown in studies to be incompetent in achieving satisfactory restoration and maintaining stability, and to bring about the risks of pseudoarticulation formation and neurological deterioration.^5,23–25^ Consequently, the majority of scholars recommend surgical treatment for AS cervical spine fracture.^26–28^ Our patients all met the requirements for operation with SLIC scores ranging from 5 to 9. Operative methods included anterior cervical approach, posterior cervical approach, and combined surgery of posterior–anterior or anterior–posterior cervical approach. Currently, the controversy over the selection of surgical methods is still intense. Most researchers suggest that as 3-column fracture, AS cervical spine fracture needs to be treated by combined surgery of posterior–anterior or anterior–posterior 360° fusion because anterior approach alone can not fulfill a completely effective replacement and this fracture tends to develop complications of screw loosening and plate displacement.^8,29^ Some other scholars, however, insist that simple posterior approach can achieve decompression, restoration, and stable fixation and fusion, making added anterior approach unnecessary. Nonetheless, the operators in this study selected personalized methods for each patient in accordance with the improved classification standards for AS cervical spine fracture. In I type patients, 6 people were treated with anterior approach, 5 with posterior approach, and 1 with anterior–posterior combined surgery. As for II type patients, posterior approach was used on 2 patients and posterior–anterior combined surgery on 3 cases. Among III type patients, 2, 2, and 4 of them received posterior approach, anterior–posterior combined surgery, and posterior–anterior combined surgery, respectively. Ideal effects were achieved in 9 cases receiving posterior approach and in 10 cases undergoing posterior–anterior or anterior–posterior combined surgery. Two patients, showing screw loosening and plate displacement in reexamination, received posterior revision surgeries in our hospital 3 and 5 months later, respectively, after anterior short segments fixation and fusion in other hospitals. The other 4 cases of anterior long segments fixation were in a state of bone grafting fusion by the end of the follow-up, and showed no distinct loosening or dislocation for internal fixation. During operation, intervertebral discs and cartilage endplate adjacent to fractures should be adopted preferentially to perform bone grafting fusion after scraping, avoiding cross-segment fixation following corpectomy.

AS cervical spine fracture is accompanied by a high incidence rate of complications, of which progressive neurological deterioration and pulmonary infections are the most common. The general reasons for the former contain delayed diagnosis after slight neck trauma, insecure fixation, and secondary damage of cervical spinal cord neurological function caused by improper preoperative traction. Respiration failure resulting from pulmonary infections is a main cause of death in AS cervical spine fracture patients, which is related to the following factors: lesions involve thoracic vertebrae and ribs, whereas constant thorax is limited to expansion, leading to reduced lung functions; as for patients with severe cervical ante flexion, they may have a difficulty in breathing due to airway under prolonged pressure; diaphragm may lose innervations after high cervical spine core was injured, contributing to substantially decreased capability of respiration, self-determined cough, and expectoration. Therapeutic measures about it contain the following aspects: tracheotomy can be performed if necessary for assisted breathing with ventilator based on the assessment of patients’ ability for respiration, self-determined cough, and expectoration; patients receive airway atomization to eliminate phlegm, and sputum suction through regular turning over and patting back or through fiberobronchoscopy when necessary; sensitive antibiotics are used in anti-infective treatments; and patients get training on their capacities in self-determined breathing, cough, and expectoration so as to generally cast off the ventilators with incision blocking. Among 8 cases (32%) developing pulmonary infections, 4 patients underwent tracheotomy during hospitalization and met successful incision blocking when leaving. One of them, however, died of respiratory failure because of pulmonary reinfection 2 months after leaving hospital.

In conclusion, AS cervical spine fracture, an unstable fracture, should be treated through surgery, and personalized selection of operative methods should be adopted for all patients according to their fracture characteristics. Our patients were all treated using corresponding surgical methods based on the modified classification of AS cervical spine fracture, which produced satisfactory effects. Different from general traumatic cervical spine fractures, AS cervical spine fracture shows a high incidence rate both in spinal neurological damage and complications. Furthermore, respiratory failure resulting from pulmonary infections is a main cause of death in AS cervical spine fracture patients. Therefore, sufficient attention should be paid to this disease in clinical treatments.
